# Ebola vaccine uptake and attitudes among healthcare workers in North Kivu, Democratic Republic of the Congo, 2021

**DOI:** 10.3389/fpubh.2023.1080700

**Published:** 2023-07-25

**Authors:** Reena H. Doshi, Stephanie C. Garbern, Shibani Kulkarni, Shiromi M. Perera, Monica K. Fleming, Rigobert Fraterne Muhayangabo, Arsene Balene Ombeni, Dieula Delissaint Tchoualeu, Ruth Kallay, Elizabeth Song, Jasmine Powell, Monique Gainey, Bailey Glenn, Ruffin Mitume Mutumwa, Stephane Hans Bateyi Mustafa, Giulia Earle-Richardson, Hongjiang Gao, Neetu Abad, Gnakub Norbert Soke, David L. Fitter, Terri B. Hyde, Dimitri Prybylski, Adam C. Levine, Mohamed F. Jalloh, Eta Ngole Mbong

**Affiliations:** ^1^Global Immunization Division, Centers for Disease Control and Prevention, Atlanta, GA, United States; ^2^Department of Emergency Medicine, Brown University, Providence, RI, United States; ^3^International Medical Corps, Washington, DC, United States; ^4^International Medical Corps, Goma, Democratic Republic of Congo; ^5^Brown University, Providence, RI, United States; ^6^Rhode Island Hospital, Providence, RI, United States; ^7^James A. Ferguson Infectious Disease Program, Baltimore, MD, United States; ^8^Expanded Programme on Immunization, Goma, Democratic Republic of Congo; ^9^National Center for Emerging and Zoonotic Diseases, Centers for Disease Control and Prevention, Atlanta, GA, United States; ^10^Division of Global Health Protection, Centers for Disease Control and Prevention, Kinshasa, Democratic Republic of Congo; ^11^Division of Global HIV and TB, Centers for Disease Control and Prevention, Atlanta, GA, United States

**Keywords:** Ebola, Ebola vaccine, vaccine hesitancy, vaccine acceptance, Democratic Republic of the Congo, Ebola virus disease (EVD), vaccines

## Abstract

**Introduction:**

During the 2018–2020 Ebola virus disease (EVD) outbreak in the eastern part of the Democratic Republic of the Congo (DRC), prevention and control measures, such as Ebola vaccination were challenging by community mistrust. We aimed to understand perceptions regarding Ebola vaccination and identify determinants of Ebola vaccine uptake among HCWs.

**Methods:**

In March 2021, we conducted a cross-sectional survey among 438 HCWs from 100 randomly selected health facilities in three health zones (Butembo, Beni, Mabalako) affected by the 10th EVD outbreak in North Kivu, DRC. HCWs were eligible if they were ≥ 18 years and were working in a health facility during the outbreak. We used survey logistic regression to assess correlates of first-offer uptake (i.e., having received the vaccine the first time it was offered vs. after subsequent offers).

**Results:**

Of the 438 HCWs enrolled in the study, 420 (95.8%) reported that they were eligible and offered an Ebola vaccine. Among those offered vaccination, self-reported uptake of the Ebola vaccine was 99.0% (95% confidence interval (CI) [98.5–99.4]), but first-offer uptake was 70.2% (95% CI [67.1, 73.5]). Nearly all HCWs (94.3%; 95% CI [92.7–95.5]) perceived themselves to be at risk of contracting EVD. The most common concern was that the vaccine would cause side effects (65.7%; 95% CI [61.4–69.7]). In the multivariable analysis, mistrust of the vaccine source or how the vaccine was produced decreased the odds of first-time uptake.

**Discussion:**

Overall uptake of the Ebola vaccine was high among HCWs, but uptake at the first offer was substantially lower, which was associated with mistrust of the vaccine source. Future Ebola vaccination efforts should plan to make repeated vaccination offers to HCWs and address their underlying mistrust in the vaccines, which can, in turn, improve community uptake.

## Introduction

The 10th outbreak (1 August 2018–25 July 2020) of Ebola virus disease (EVD) in the Democratic Republic of the Congo (DRC) affected three provinces and was centered in North Kivu and Ituri. This outbreak became the second largest EVD outbreak globally after the 2014–2016 West Africa EVD outbreak. (1) It was officially declared over after almost 2 years, and 3,481 cases (3,323 confirmed, 158 probable) and 2,299 deaths (2). Unlike previous EVD outbreaks in DRC, this outbreak was unprecedented in terms of the geographical scope, the number of cases, and duration. The region’s long history of armed conflict presented obstacles for response teams to rapidly identify and isolate suspect cases, trace contacts, conduct risk communication and community engagement activities, provide specialized medical care, and administer vaccines (3–5).

Of the confirmed EVD cases during this outbreak in DRC, 5% were health care workers (HCWs) and 44% died (1). Health care workers are at increased risk of EVD given their close physical contact with patients and their bodily fluids (6, 7). Thus, the Strategic Advisory Group of Experts on Immunization (SAGE) recommends Ebola vaccination for HCWs and other contacts during an active EVD outbreak (8, 9). Vaccination reduces the risk of EVD and may prevent nosocomial transmission, especially in the context of poor adherence to infection and prevention control methods (10, 11).

Since 2018, vaccination with the ERVEBO® (rVSVΔG-ZEBOV-GP) vaccine has been an integral part of EVD outbreak response in DRC, administered under a compassionate use/expanded access protocol (12). The vaccine has been shown to be safe and effective against *Zaire ebolavirus* in Phase 3 trials and was further confirmed through data collected in DRC under the compassionate use protocol. The vaccine has since been approved for use in several countries (13). In 2019, the vaccine was licensed by the European Medicines Agency and the United States Food and Drug Administration, though the use of the licensed product in recent EVD outbreaks had been challenged by supply constraints (14).

Vaccine hesitancy, or the state of indecision and uncertainty about vaccination, has become an increasing public health threat globally (15, 16). Vaccine hesitancy is complex, context-specific, and varies by time, place, and type of vaccine and is influenced by numerous factors including, confidence in the vaccine safety and efficacy; public knowledge and awareness; and religious, cultural, gender, or socioeconomic factors (17, 18). Further, social determinants, such as the ability to take time off from work and travel to vaccination sites, and ability to miss work due to side effects, place additional constraints on individuals who might otherwise accept vaccination. Studies from the 2014–2016 West African EVD outbreak revealed multiple drivers of Ebola vaccine acceptance, including perceived risk of Ebola, altruistic desires to prevent Ebola transmission, trust, or distrust of those offering the vaccines, and fear of side effects (19–26). In North Kivu, dissatisfaction and mistrust, of the government and EVD response teams was widespread (27). Challenges in gaining community confidence in Ebola vaccination and other preventive measures severely undermined response activities (4, 27). Community feedback collected during the outbreak, which included HCWs, found that inadequate knowledge and politicization of EVD, skepticism concerning vaccination efficacy and necessity, perceptions of unfairness around prioritizing contacts for vaccination, safety concerns, and beliefs about international organizations harboring ulterior motives were barriers to Ebola vaccination and undermined vaccine confidence (3, 5, 28).

There is little published data HCWs perceptions and attitudes about Ebola vaccine in DRC. Understanding attitudes among HCWs is not only important because they are at high risk for EVD infection, but also because HCWs can play an important role in encouraging others in the community to be vaccinated. There is evidence that vaccination attitudes among HCWs predict their vaccine uptake and intention to recommend vaccine in both routine health care settings and during health emergencies (27, 29–31).

An improved understanding of the drivers of timely Ebola vaccination uptake among HCWs is needed to better inform new vaccine introduction efforts, especially during emergencies. This survey aimed to understand the perceptions about Ebola vaccination, measure Ebola vaccine uptake, and identify determinants of Ebola vaccine uptake among HCWs, who were offered the vaccine during the 2018–2020 EVD outbreak.

## Methods

### Survey setting

**Figure 1:**
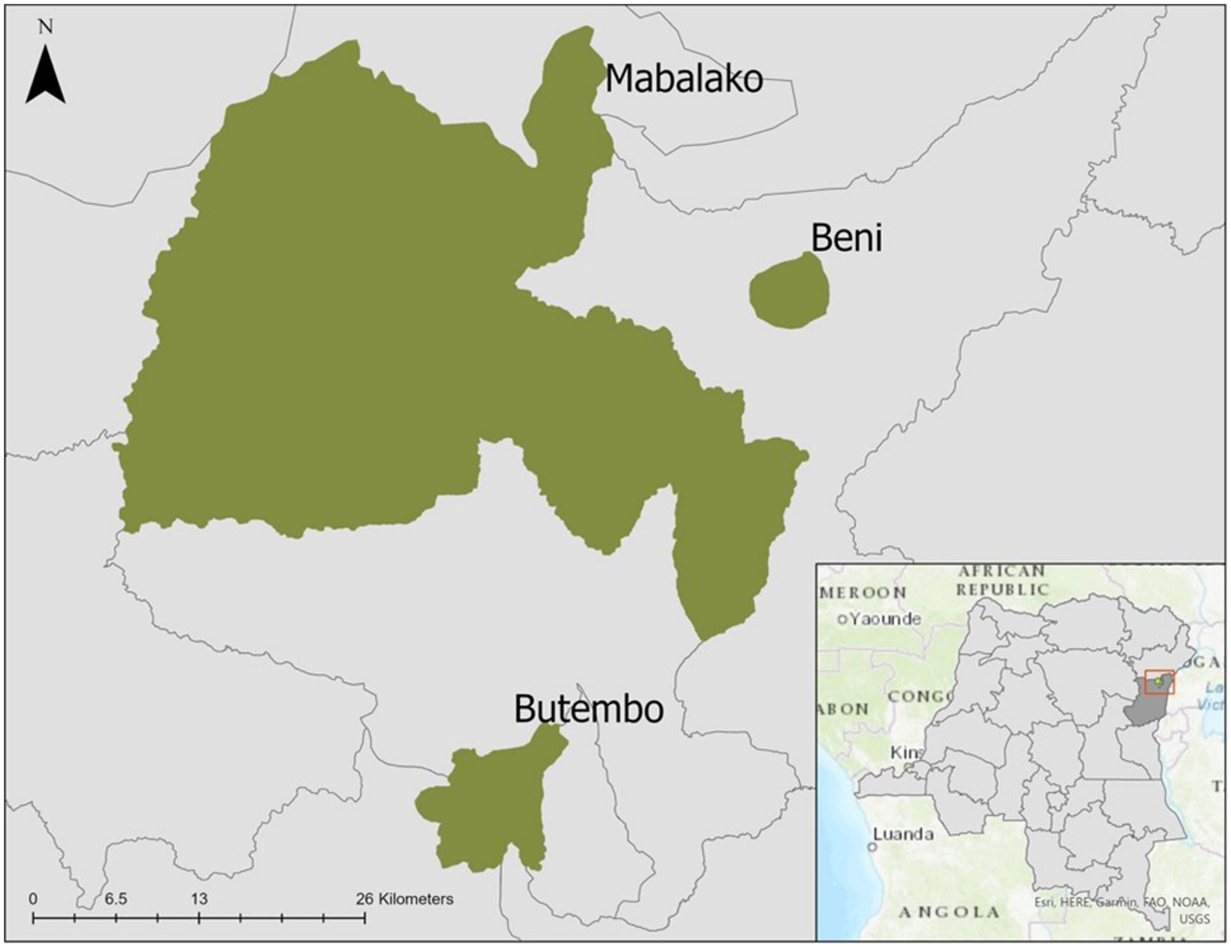
Map of selected health zones in North Kivu (Beni, Butembo, Mabalako), Democratic Republic of the Congo, 2021.

North Kivu Province is in eastern DRC and forms part of the country’s borders with Uganda and Rwanda (Figure 1). The region has more than four decades of conflict and insecurity, the presence of multiple armed groups, large-scale population displacements, poverty, limited access to essential healthcare services, and distrust of both the government and foreigners (4, 5, 32). During the 10th EVD outbreak, vaccination with investigational (unlicensed) doses of the ERVEBO (rVSVΔG-ZEBOV-GP) vaccine was initiated on 8 August 2018, under an “expanded access/compassionate use” protocol, requiring informed consent. Ring vaccination was the primary vaccination strategy, but HCWs were also offered vaccination if they worked in a health facility in active outbreak areas (33, 34). Pregnant and lactating women were initially not eligible for vaccination, but the protocol was revised in June 2019 to include these women due to increasing EVD cases (35).

### Survey design and sampling

In March 2021, we conducted a cross-sectional survey among HCWs in three health zones in North Kivu Province, DRC. Butembo, Beni and Malabako health zones were selected due to their high EVD cases counts and reports of community resistance to response activities during the 10th outbreak in DRC. We used a stratified one stage cluster design assuming a design effect of 2, halfwidth of 95% confidence interval (CI) equal to 7.5%. A random sample of 100 health facilities was chosen from a sampling frame of 187 facilities (81 in Butembo, 79 in Beni, and 27 in Mabalako) that were operational during the outbreak and provided health services to the local communities. The sampling frame included primary health facilities (e.g., health posts, dispensaries, health centers) generally run by community HCW and nurses, and secondary health facilities (e.g., hospitals, referral medical centers where the population might receive more specialized care). Both public and private health facilities were included in the sample.

### Inclusion criteria

HCW were defined as anyone working at a health facility at the time of data collection and included both clinical and support roles (including administrative, cleaners, hygienists). A line list of all staff at each selected facility was created and HCWs were invited to participate if they indicated they had been working in any health facility during the outbreak (August 2018–July 2020) and were at least 18 years of age. A second visit to the health facility was made to enroll any absent the first day. In total, 445 HCWs were screened and 440 were eligible; two HCWs declined to participate.

### Survey instrument

A structured questionnaire was developed that consisted of the following topic areas: survey eligibility, participant demographics, and knowledge, attitudes and beliefs about the Ebola vaccine and the vaccination program. These questions were mapped to five areas that were expected to influence vaccine uptake: perceived risk, communications (including rumors, misinformation, community engagement), societal factors (including religion, community leaders, government, security concerns), health systems (e.g., vaccination teams, geography, logistics) and vaccine specific factors (e.g., safety and efficacy). The topic areas were selected based on review of the literature, review of community feedback produced during the outbreak, and knowledge of the region. To assess general vaccine confidence, we utilized a series of six questions that had been psychometrically validated in the context of childhood vaccination in Sierra Leone (36). A draft survey was pilot tested in a selected community near the training site (the pilot data were not included in the final analysis).

### Data collection

Face to face interviews were conducted in March 2021 at the health facilities. Trained multi-lingual interviewers were recruited locally and received a four-day training. Interviewers worked in pairs (male and female) and interviewed participants who were of the same sex as themselves. Participation was anonymous, voluntary, and uncompensated. Interviewers administered the survey in French for francophone participants and performed on-the-spot translations in Swahili, as necessary per training guidance. Responses from the participants were recorded by interviewers on mobile devices pre-programmed with KoBoCollect (Kobo, Inc.) All data were uploaded daily with nightly back-ups to the KoBo Toolbox server. Data collection teams were required to follow COVID-19 mitigation measures, including wearing a mask, using hand sanitizer, and social distancing where possible.

### Descriptive analysis

To describe Ebola vaccine uptake and perceptions about Ebola vaccination, we calculated frequencies of survey responses related to vaccine uptake, attitudes, beliefs and vaccine concerns. Vaccine safety, efficacy, and trust were measured on a 5-point bipolar Likert scale in the questionnaire (ranging from strongly agree to strongly disagree). Point estimates and 95% were calculated using STATA’s “svy: tabulate procedure.”

### Multivariable modeling

Logistic regression was used to examine correlates associated with HCW acceptance or delay of the vaccine. The outcome variable was HCW self-reporting accepting the Ebola vaccine the first time it was offered vs. those who accepted at the second or later offers. We selected variables for inclusion in the model based on expert consensus and taking into account the constructs in the Health Belief Model (37). The “svyset” command in STATA was used to specify clustering at the health facility level, applying sampling weights to account for the cluster survey design. Stratification of health facilities as primary or secondary facilities were included in the survey sampling design.

The sample was limited to 415 participants that were eligible for the Ebola vaccine, offered vaccination, recalled the number of times they were offered the vaccine, and took the vaccine at the first offer or on subsequent offers. To identify factors significantly associated with vaccine uptake, three composite vaccine confidence variables (safety, efficacy, and trust) were created by first dichotomizing the 5-point Likert scale by classifying “strongly agree,” and “agree” as “yes” and “neither agree or disagree,” “disagree,” and “strongly disagree” as “no.” For general vaccine confidence, we computed a composite score, using six items (Supplementary Table 1) that have been psychometrically validated in the context of childhood vaccination in Sierra Leone (36). Each question had a scale of 0–4 corresponding to low-high vaccine acceptance and the total composite score was then categorized as low (<25th percentile), medium (25–75th percentile), or high vaccine (>75th percentile). The following variables were included in the model: sex, age, highest education level attained, religion’s influence on health decisions, perceived risk of contracting Ebola during the outbreak, perception that Ebola can be prevented with a vaccine and the perception of the Ebola vaccine as having severe side effects, trust in vaccine source/how it was produced and general vaccine acceptance. We conducted a sensitivity analysis, substituting the variable of the current perceived risk of Ebola (“I think I am now at risk of contracting Ebola”) as a binary variable (strongly agree and agree coded as yes and neutral, disagree, and strongly disagree as no) instead of the recalled perceived risk of contracting Ebola during the outbreak in regression analysis. All analyses were performed using Stata version 16 (Stata Corp; College Station, United States).

### Ethical considerations

The University of Kinshasa School of Public Health Ethics Committee approved the survey (protocol approval #203-2020). The assessment was determined to be a non-research public health activity by CDC. The purpose of the assessment was explained to all participants with additional details provided on an information sheet in the local language. Verbal informed consent was obtained and documented electronically on the data collection tool by trained research staff, due to low literacy rates and the need to limit physical contact during the pandemic.

## Results

Of the 438 participating HCW, the median age was 35 years (IQR 29–42) with females comprising 53.7% of the sample. Nearly all respondents (90.1%) had at least some secondary school education. The most reported occupations were nurse (47.7%), hygienist (17.4%), and administrator (10.5%). Overall, 40.2% of HCWs participated in the outbreak response in DRC (Table 1).

**Table 1:** Characteristics of surveyed health care workers (*N* = 438), North Kivu, Democratic Republic of the Congo, 2021.

Characteristic	*n*	%^**^
Age category (years)^*^		
18–24	45	10.3
25–39	235	53.7
40–54	133	30.3
≥55	25	5.7
Health zone		
Beni	167	38.1
Butembo	172	39.3
Mabalako	99	22.6
Sex		
Male	203	46.3
Female	235	53.7
Highest education level		
None	10	2.3
Primary school	33	7.5
Secondary school	178	40.6
University or Higher Institute	215	49.1
Declined to Respond	2	0.5
Religion		
Catholic	227	51.8
Protestant/Evangelical/Pentecostal	196	44.7
Muslim	1	0.2
Other^	14	3.2
Influence of faith on decisions including health		
No influence	152	34.7
Influences some decisions	170	38.8
Influences all decisions	114	26.0
Declined to respond	2	0.5
Health facility level		
Primary (health post, clinic, dispensary)	246	56.2
Secondary (hospital, medical center, referral center)	192	43.8
Occupation		
Nurse	209	47.7
Doctor	20	4.6
Administrator	46	10.5
Hygienist	76	17.4
Midwife	14	3.2
Lab Technician	25	5.7
Physiotherapist	3	0.7
Medical/Nursing Student	19	4.3
Data Manager	10	2.3
Pharmacist	4	0.9
Other†	12	2.7
Worked in the 10th EVD outbreak		
Yes	176	40.2
No	262	59.8

* Age median [interquartile range], 35 [29–42] years.

** Unweighted percentages.

^^^Other religions: Adventist, Anglican, Jehovah’s Witness, Zionist, Animist. ^†^Other occupations: Lab assistant, receptionist, pharmacy worker, secretary.

### Outbreak experiences

Nearly all HCWs, 94.3% [95% Confidence Interval: 92.7%–95.5%] perceived themselves to be at some risk of contracting EVD during the tenth outbreak. Approximately half 51.8% [95% CI: 48.8%–54.8] reported they had been in contact with someone with EVD during the outbreak and 2.7% [95% CI: 2.0–3.7] reported they had been diagnosed with EVD.

### Perceptions of EVD and Ebola vaccines

The majority of HCWs felt that Ebola is a potentially fatal disease, with 76.9% ([95% CI: 74.5–79.2]) strongly agreeing and 22.2% ([95% CI: 19.9–24.5]) agreeing (Table 2). Most respondents had favorable beliefs regarding the vaccines’ efficacy with 56.6%; ([95% CI: 53.5–59.7]) strongly agreeing and 36.8% (95% CI: [33.8–39.8]) agreeing that the vaccine is needed to prevent disease spread during outbreaks, and 44.1% (95% CI: [40.8–47.4]) strongly agreeing and 41.8% (95% CI: [39.0–44.6]) agreeing that the vaccine reduces disease severity. Some HCWs did not have confidence in the vaccine with 5.7% (95% CI: [4.7–7.0]) strongly agreeing and 21.0% (95% CI: 18.9%–23.3%) agreeing that they did not trust the Ebola vaccine source or how it was produced (Table 2). Among all respondents, 64.6% (95% CI: [61.4–67.7]) felt that the Ebola vaccine should be mandatory for HCWs (data not shown in table).

**Table 2:** Perceptions of Ebola virus disease and the Ebola vaccine among surveyed health care workers, North Kivu, Democratic Republic of the Congo, 2021.

Item	Strongly agree	Agree	Neither agree nor disagree	Disagree	Strongly disagree	Unsure/declined
*n* (%) [95% CI]						
(*N* = 438)						
Ebola is a serious disease that can kill me	337 (76.9) [74.5–79.2]	97 (22.2) [19.9–24.5]	2 (0.5) [0.2–0.9]	0	0	2 (0.5) [0.2–0.9]
Ebola vaccine is needed to prevent disease spread during an outbreak	248 (56.6) [53.5–59.7]	161 (36.8) [33.8–39.8]	10 (2.3) [1.7–3.1]	7 (1.6) [1.1–2.4]	7 (1.6) [1.1–2.4]	5 (1.1) [0.8–1.7]
The vaccine reduces disease severity	193 (44.1) [40.8–47.4]	183 (41.8) [39.0–44.6]	14 (3.2) [2.5–4.1]	22 (5.0) [4.0–6.3]	11 (2.5) [1.8–3.4]	15 (3.4) [2.6–4.4]
The vaccine has severe side effects	69 (15.8) [13.9–17.9]	197 (45) [42.1–47.8]	36 (8.2) [7.0–9.6]	102 (23.3) [21.0–25.7]	24 (5.5) [4.2–7.1]	10 (2.3) [1.7–3.0]
I think I am now at risk of contracting Ebola	57 (13.0) [10.7–15.8]	167 (38.1) [35.4–40.9]	46 (10.5) [8.6–12.8]	109 (24.9) [22.4–27.5]	38 (8.7) [35.4–40.9]	21 (4.8) [3.9–5.9]
I wanted to be vaccinated when the vaccine was available in my community	149 (34.0) [30.8–37.4]	216 (49.3) [45.9–52.7]	15 (3.4) [2.6–4.5]	42 (9.6) [8.0–11.4]	11 (2.5) [1.8–3.4]	5 (1.1) [0.7–1.8]
Getting vaccinated makes me feel I do not need to take other precautions to protect myself against Ebola	10 (2.3) [1.6–3.2]	25 (5.7) [4.5–7.2]	11 (2.5) [1.9–3.3]	176 (40.2) [37.2–43.2]	210 (47.9) [44.8–51.1]	6 (1.4) [0.9–2.1]
Many people were vaccinated in my community	167 (38.1) [34.9–41.5]	225 (51.4) [48.2–54.5]	8 (1.8) [1.3–2.5]	18 (4.1) [3.3–5.2]	13 (3) [2.3–3.9]	7 (1.6) [1.1–2.3]
I did not trust the local team that was offering the vaccine	27 (6.2) [4.8–7.8]	87 (19.9) [17.6–22.3]	48 (11.0) [9.0–13.2]	194 (44.3) [41.3–47.3]	70 (16.0) [14.1–18.1]	12 (2.7) [1.9–4.0]
I did not trust the source or how the vaccine was produced	25 (5.7) [4.7–7.0]	92 (21.0) [18.9–23.3]	73 (16.7) [14.5–19.0]	157 (35.8) [33.2–38.6]	68 (15.5) [13.2–18.1]	23 (5.3) [4.1–6.6]
The security situation in my area prevents me from getting vaccinated or seeking other health services	28 (6.4) [5.1–8.0]	54 (12.3) [10.4–14.6]	43 (9.8) [8.2–11.7]	191 (43.6) [40.8–46.5]	103 (23.5) [20.6–26.7]	19 (4.3) [3.5–5.4]
I do not trust the government to make decisions about vaccines	23 (5.3) [4.2–6.5]	93 (21.2) [19.0–23.6]	86 (19.6) [17.4–22.0]	133 (30.4) [28.1–32.7]	87 (19.9) [17.6–22.3]	16 (3.7) [2.9–4.6]

* All responses were based on a 5 point Likert scale.

### Ebola vaccine eligibility and uptake

Among the 438 enrolled respondents, 420 (95.9%; 95% CI: [94.7–96.8]) reported that they were eligible for and offered the Ebola vaccine. Of the 420 respondents who were offered vaccination, 99.0% (95% CI: [98.5–99.4]) self-reported that they took the vaccine (Table 3). Among those who took the Ebola vaccine (*n* = 416), uptake at the first offer was 70.2% (95% CI: [67.1–73.5]) compared to 12.3% (95% CI: [10.4–14.4]) at the second offer, 7% (95% CI: 5.6–8.7) at the third offer, and 10.3% (95% CI: [8.6–12.4]) at the fourth offer or later (Table 3).

**Table 3:** Ebola vaccination status and the number of offers prior to vaccine receipt among vaccinated healthcare workers, North Kivu, Democratic Republic of the Congo, 2021.

Ebola vaccine eligibility and vaccination status	*n*	% [95% CI]
Eligibility and vaccine offers	*N* = 438	
Eligible and offered opportunity to receive vaccine	420	95.9 [94.7–96.8]
Ineligible or not offered vaccine^*^	18	4.1 [3.2–5.3]
Vaccine uptake^+^	*N* = 420	
Vaccinated	416	99.0 [98.5–99.4]
Unvaccinated^†^	4	1.0 [0.6–1.5]
Timeliness of Ebola vaccine uptake	*N* = 416	
Vaccinated at first offer	292	70.2 [66.9–73.3]
Vaccinated at second offer	51	12.3 [10.3–14.4]
Vaccinated at third offer	29	7.0 [5.6–8.7]
Vaccinated at fourth offer or later	43	10.3 [8.6–12.4]
Do not recall timing of uptake against the offering	1	0.2 [0.1–0.6]

* Not offered or informed they were ineligible as per patient’s report. Specific reasons for which patients were informed they were ineligible were not solicited in the survey.

^†^Of the four HCWs who were eligible but declined the vaccine, three reported they declined due to being pregnant or breastfeeding, and one due to having an underlying chronic disease. ^+^Based on self-reported vaccination status.

### Vaccine concerns

Nearly half (48.3%; [95% CI: 45.7–51]) of the vaccinated participants (*n* = 416) reported they had concerns about the vaccine when they received it (Table 4). The most common concerns were that the vaccine would cause side effects (65.7%; [95% CI: 61.4–69.7]), or death (48.5%; [95% CI:44.6–52]), cause EVD (29.4%; [95% CI: 26.4–32.5%]), and cause infertility (33.3%; [95% CI: 29.3–37.4]). Fewer were concerned that the vaccine was not effective at preventing EVD (17.9%; [95% CI: 14.8–21.5]) or had a lack of trust in the vaccine manufacturer (12.9%; [95% CI 10.7–15.6]) and the process used to make the vaccine (4.5%; [95% CI: 3.3–6.0]). In an unadjusted analysis (data not shown), HCWs with concerns about the vaccine had lower odds of first-offer vaccine acceptance (OR 0.26; [95% CI: 0.14–0.50]) vs. those without concerns.

**Table 4:** Ebola vaccine concerns at the time of vaccination among health care workers (*N* = 416), North Kivu, Democratic Republic of the Congo, 2021.

	*n*	%, [95% CI]
Did you have concerns about the vaccine when you received it?	*N* = 416	
Yes	201	48.3 [45.7–51.0]
No	215	51.7 [49.0–54.3]
If yes, what concerns did you have?	*N* = 201^*^	
It may cause Ebola virus disease	59	29.4 [26.4–32.5]
It may cause death	97	48.3 [44.6–52.0]
It may cause side effects like muscle aches and body pain.	132	65.7 [61.4–69.7]
It may cause infertility or sexual/weakness	67	33.3 [29.4–37.4]
It cannot prevent Ebola	36	17.9 [14.8–21.5]
Lack of trust in vaccine manufacturer	26	12.9 [10.7–15.6]
Lack of trust in the process used to make the vaccine	9	4.5 [3.3–6.0]
Lack of trust in the team offering the vaccine	7	3.5 [2.2–5.5]
Lack of trust in the health system	2	1.0 [0.5–1.9]

* Participants could indicate multiple responses.

### General vaccine confidence

Participants had overall positive attitudes toward vaccines in general with 88.6% (95% CI: [86.8–90.2]) reporting they “very much” or “somewhat” agreed that vaccines are good and 81.7%; (95% CI: [79.5–83.8]) “very much” or “somewhat” agreed that vaccines are safe (Supplementary Table 1). However, only 10.3% (95% [CI: 8.5–12.3]) reported that the community spoke positively about vaccines in general, while 24.9% (95% [CI: 22.5–27.5]) reported the community spoke negatively.

### Multivariable analysis

In the multivariable analysis, older age (adjusted Odds Ratio (aOR) 1.14 [95% CI: 1.02–1.28]), having been an Ebola response team member (aOR 1.41 [95% CI: 1.06–1.87]), perception that Ebola can be prevented with a vaccine (aOR 1.92 [95% CI: 1.47–2.51]) and having high general vaccine confidence (aOR 2.33 [95% CI: 1.70–3.21]) were all associated with a higher odds of first-offer vaccine uptake. Participants who expressed mistrust of the vaccine source or how the vaccine was produced were found to have lower odds of first-offer vaccine uptake (aOR 0.38 [95% CI: 0.30–0.47]). Those with perceived risk of contracting EVD during the outbreak (aOR 0.43 [95% CI: 0.22–0.87]) were negatively associated with first-offer vaccine uptake in the primary analysis, however, in sensitivity analyses, current perceived risk of contracting EVD was found to have no association with first-offer vs. later offer uptake (aOR 0.86 [95% CI: 0.67–1.10]). All other covariates in the model, including sex, educational level, religion’s influence, and belief that the Ebola vaccine has severe side effects, were not significantly associated with first-offer uptake (Table 5).

**Table 5:** Correlates of Ebola vaccine uptake at the first offer vs. subsequent offers among vaccinated health care workers, North Kivu, Democratic Republic of the Congo, 2021 (*N* = 415).

	First Offer *n* (%) *N* = 292	Multiple Offers n (%) *N* = 123	OR [95% CI]	aOR [95% CI]
Sex				
Male	144 (49.3)	54 (43.9)	*Reference*	*Reference*
Female	148 (50.7)	69 (56.1)	0.80 [0.66–0.98]	1.02 [0.82–1.29]
Age (years), median [IQR]	36 (30–43)	34 (27–41)	1.24 [1.10–1.39]	1.14 [1.02–1.29]^
Highest education attained				
Primary or lower	27 (9.3)	12 (9.8)	*Reference*	*Reference*
Secondary	119 (40.8)	46 (37.4)	1.15 [0.77–1.71]	1.25 [0.79–1.98]
University or higher	144 (49.3)	65 (52.9)	0.98 [0.69–1.40]	0.87 [0.58–1.31]
Missing/declined	2 (0.7)	0 (0)	n/a	n/a
Influence of religion on vaccination				
No influence	101 (34.6)	39 (31.7)	*Reference*	*Reference*
Influences some decisions	119 (40.8)	44 (35.8)	1.04 [0.74–1.48]	1.28 [0.90–1.82]
Influences all decisions	72 (24.7)	38 (30.9)	0.73 [0.51–1.05]	0.78 [0.58–1.31]
Missing/declined	0 (0)	2 (1.6)	n/a	n/a
Ebola response team member				
No	160 (54.8)	81 (65.9)	*Reference*	*Reference*
Yes	132 (45.2)	42 (34.2)	1.59 [1.24–2.04]	1.41 [1.06–1.87]
Perceived risk of EVD during outbreak				
No	16 (5.5)	3 (2.4)	*Reference*	*Reference*
Yes	276 (94.5)	120 (97.6)	0.43 [0.22–0.85]	0.45 [0.22–0.89]
Ebola can be prevented with vaccine^*^				
No	16 (5.5)	3 (2.4)	*Reference*	*Reference*
Yes	276 (94.5)	120 (97.6)	2.00 [1.54–2.59]	1.92 [1.47–2.51]
Ebola vaccine has severe side effects^*^				
No	124 (42.5)	42 (34.2)	*Reference*	*Reference*
Yes	168 (57.5)	81 (65.8)	0.70 [0.54–0.91]	0.85 [0.66–1.10]
Mistrust of vaccine source or how it was produced*				
No	235 (80.5)	73 (59.4)	*Reference*	*Reference*
Yes	57 (19.5)	50 (40.7)	0.35 [0.28–0.44]	0.38 [0.30–0.47]
General vaccine confidence^**+**^				
Low	51 (17.5)	38 (30.9)	*Reference*	*Reference*
Medium	130 (44.5)	57 (46.3)	1.70 [1.28–2.26]	1.31 [0.97–1.76]
High	111 (38.0)	28 (22.8)	2.95 [2.11–4.13]	2.34 [1.70–3.21]

Only vaccinated participants who recalled the number of offers prior to acceptance were included in the 415. aOR, adjusted odds ratio from mixed-effects logistic regression model for first-offer vaccine uptake.

* Recoded from a Likert scale to yes or no. ^^^10 years intervals. ^+^A composite score was computed using six items, each question had a scale of 0–4 (36). Each question had a scale of 0–4 corresponding to low-high vaccine acceptance, the total composite score was then categorized as low (<25th percentile), medium (25–75th percentile), or high vaccine (>75th percentile).

## Discussion

Our findings revealed high uptake of Ebola vaccine among HCWs in three health zones heavily affected by the 10th EVD outbreak in DRC. While the majority of HCWs considered EVD to be a serious disease that could kill them, almost a third of the eligible HCWs delayed vaccination. Being a response worker, the perception that Ebola could be prevented with a vaccine and having higher general confidence in vaccines were all associated with higher odds of accepting the vaccine on first offer, while mistrust in the vaccine source was negatively associated with vaccine uptake at the first offer. Taken together, our findings imply that we must address underlying trust in the vaccine and that uptake is enhanced with multiple vaccination offers. Vaccine outreach activities with strong risk communication and community engagement efforts that improve the timeliness of EVD vaccination among HCWs may have the dual benefit of protecting the frontline workforce and increasing the likelihood that HCWs may encourage others to take the vaccine. Early efforts to target this group can build vaccine confidence, which is important in controlling outbreaks of vaccine preventable diseases.

The high level of vaccine acceptance in this HCW population is consistent with prior research and might be explained by several reasons (24, 38). HCWs likely had multiple opportunities to get vaccinated given that Beni, Butembo, and Mabalako were all frequent epicenters during the outbreak and vaccination teams were likely present in the communities at multiple points during the 22-month outbreak. Many HCWs were eligible and likely offered vaccination multiple times, especially if they were working in a facility at the time of the outbreak, or if they were part of a ring (i.e., a contact or contact of contact of an EVD case). Strong encouragement from response workers or the health facilities and potentially the fear of job loss, might also explain the high vaccination rate.

Despite high acceptance, mixed perceptions regarding the vaccine were common, with nearly half of respondents reporting that they had concerns about the vaccine, including vaccine side effects, vaccine safety, and vaccine effectiveness. Fear of side effects and lack of confidence in vaccine effectiveness has been associated with lower vaccine acceptance (39) and many of the vaccine side effects overlap with EVD symptoms, which may have increased fear (40). Many of these concerns were communicated to individuals during the informed consent process, however only those that agreed to take the vaccine were consented.

While almost all HCWs in this sample were eventually vaccinated, almost 30% indicated that they did not take the Ebola vaccine the first time it was offered, with about 10% only accepting after 4 or more offers. Circulating rumors and misinformation on social media and throughout the local community may have contributed to the concerns, including that the experimental drugs and vaccines were brought to exterminate the local population, that the vaccine might cause death or EVD, or lead to infertility or sexual dysfunction (3, 41). We did not identify differences by age and sex, nor religion, which contrasts with past work suggesting that vaccine uptake may be closely linked to religious beliefs and perceptions from community leaders (42, 43). In the multivariable analysis, only mistrust of vaccine source was associated with delayed vaccine acceptance. Before 2018, the vaccine had not been widely deployed for use in an outbreak, and during the 10th outbreak, the vaccine was unlicensed and investigational doses were being offered under a compassionate use/expanded access protocol. This involved multiple steps, including a lengthy written informed consent process. Vaccination staff reviewed the consent form with each participant, which included information on previous safety evaluations. In addition, staff described the side effects of the vaccine and were provided with paracetamol post vaccination. In response to increasing case counts and geographic spread, the protocol was revised to include pregnant women (after the first trimester) and lactating women (9). Additionally, a fractional dose was offered to preserve vaccine supply (9) and a second vaccine, offered as a two dose regimen, was given in areas near the active outbreak (44). During the West African EVD outbreak in 2014–2016, HCWs expressed concerns about the experimental status of the vaccine (19–21, 24). Despite necessary, these changes mid-outbreak may have led to confusion and distrust in the vaccine and the process (3).

It is important to note that this EVD outbreak occurred in an active conflict zone, where low institutional trust has been documented (27). In 2019, community resistance, including attacks on response teams, HCWs, and Ebola treatment centers escalated as armed group activities also increased (45). The EVD response work was frequently undermined by misinformation from government authorities and the rapid mobilization of staff and resources for the EVD outbreak contradicted the government’s failure to protect the community from continuous violence (27, 46). Prior work from the region suggested that low levels of trust in government institutions and dissatisfaction with perceived inadequacies in the response effort, including violation of cultural burial practices have been linked to reducing adherence to EVD preventive behaviors (27).

While the Ebola vaccine acceptance was essentially ubiquitous across this group, our findings underscore the importance of efforts to engage the “moveable middle” i.e., the group that has vaccine concerns, but who is open to changing vaccination intent with additional information (47). The importance of this group has also been seen with the COVID-19 vaccine (48). With any new vaccine introduction, clear communication on potential vaccine side effects, and its safety and efficacy are crucial (13, 34, 49). Additionally, HCWs are prioritized for the Ebola vaccination owing to their nature of work; therefore, addressing vaccine confidence issues in HCWs early on can also contribute to better peer-to-peer support for improving social norms around vaccination. HCWs also act as ambassadors for trusted health information and vaccine confidence in the local community. Thus, the development of targeted strategies that empower HCWs with knowledge and ways to combat misinformation may lead to improved community uptake of the vaccine (50). Notably, general vaccine acceptance for RI was high among this sample of HCWs. While general vaccine acceptance is often correlated with acceptance for other vaccines, newer adult vaccines such as EVD or even COVID-19 may take more time to build confidence and may result in delayed acceptance (51).

More than half of the HCWs indicated that had they contact with an EVD case. HCWs serve as frontline workers during an EVD outbreak and are at higher risk for occupational exposure and transmission (6, 42). Additionally, more than half felt that Ebola vaccination should be mandatory for HCWs. There are an estimated 236,000 HCWs in 13 countries with a history of EVD outbreaks, thus preemptive vaccination for HCWs has been proposed as a strategy to avert another large-scale outbreak (52, 53). However, during the March 2021 meeting, SAGE reiterated that widespread preventive use of either Ebola vaccine was not recommended in the absence of an outbreak due to current supply constraints (54). A Gavi-funded global emergency stockpile of 500,000 doses of the Ebola vaccine is currently in progress and doses are accessible to countries in the event of an outbreak. The high vaccination acceptance among this sample of HCWs shed light on the effectiveness of prophylactic vaccination, which may be reconsidered once supply increases (8, 33). Modeling of vaccination scenarios during outbreaks suggests that prophylactic vaccination of HCWs might contribute to reductions in the outbreak size (11, 55).

Our results are subject to several limitations. While we randomly sampled from a list of health facilities in three health zones, we acknowledge that our sampling frame may not have been comprehensive of all health facilities in these zones, specifically informal facilities. Further, HCWs are a very specific group; thus, caution is required when extrapolating these results to other categories or the local communities. We were unable to quantify levels of mistrust and how it may have changed overtime. Due to the COVID-19 pandemic, data collection was delayed by 9 months. During this delay, two subsequent EVD (11th and 12th) outbreaks occurred in DRC and may have influenced our results in ways that we cannot discern. Additionally, there is a possibility of misclassification due to recall inaccuracies, particularly for questions on exposure during the outbreak and number of times the vaccine was offered. Although the survey tool was translated, piloted, and adapted to the country context, and interviewers received special training, some questions and responses may have been misinterpreted or mistranslated by the interviewers.

This work highlights the complex nature of vaccine hesitancy among HCWs in a complex environment. It also provides important information on the effectiveness of a prophylactic vaccination program for HCWs. While overall uptake of the Ebola vaccine was very high among HCWs, we found that delayed uptake was relatively common, and associated with mistrust of the vaccine source. The final decision to receive the vaccine was likely a combination of contextual, individual, community, and vaccine-specific factors, including the length and severity of the outbreak. Future Ebola vaccination efforts in this setting or other similar settings should plan to address underlying mistrust of the vaccines and to offer vaccination repeatedly. Regularly utilizing data from the novel Integrated Outbreak Analytics (IOA) approach other behavioral surveys can provide actional social science evidence to improve response activities (56). Interventions aimed at increasing trust in the vaccine by disseminating accurate vaccine information and addressing rumors may aid in increasing Ebola vaccine uptake during future outbreaks.

## Meetings

Preliminary results from this work were presented at the American Society for Tropical Medicine and Hygiene 2021 virtual annual conference.

## Data availability statement

The datasets presented in this article are not readily available because they are the property of the DRC Ministry of Health. Requests to access the datasets should be directed to the authors.

## Ethics statement

The studies involving human participants were reviewed and approved by Kinshasa School of Public Health. The Ethics Committee waived the requirement of written informed consent for participation.

## Author contributions

RD and MF: study conceptualization. MF, RD, MJ, DP, GE-R, SK, DT, NA, AL, and EM: study design. EM, RiM, AO, SH, and RuM: data collection. SG, SP, ES, JP, MG, BG, SK, MJ, and HG: data analysis and interpretation. SG, RD, SP, SK, and MJ: article drafting. GS, DF, TH, RK, AL, HG, and MJ: critical revision. All authors contributed to the article and approved the submitted version.

## Funding

Funding for this work was provided through grants from the U.S. Centers for Disease Control and Prevention (Federal Identifier: NU2GGH002058) to International Medical Corps. This work was also supported in part by the National Institute of Allergy and Infectious Diseases R25AI140490.

## Conflict of interest

The authors declare that the research was conducted in the absence of any commercial or financial relationships that could be construed as a potential conflict of interest.

## Publisher’s note

All claims expressed in this article are solely those of the authors and do not necessarily represent those of their affiliated organizations, or those of the publisher, the editors and the reviewers. Any product that may be evaluated in this article, or claim that may be made by its manufacturer, is not guaranteed or endorsed by the publisher.

## Author disclaimer

The findings and conclusions in this paper are those of the authors and do not necessarily represent the official position of the U.S. Centers for Disease Control and Prevention or International Medical Corps or any institutions that the authors are affiliated with.
