# Supplementary material for: Ebola vaccine uptake and attitudes among healthcare workers in North Kivu, Democratic Republic of the Congo, 2021
Source: Front Public Health. 2023 Jul 25;11:1080700. doi: 10.3389/fpubh.2023.1080700 (PMC10408297; doi:10.3389/fpubh.2023.1080700)
Supplement: Supplementary file 1 [file Table_1.docx]

**Supplementary Table 1.** Perceptions towards routine immunizations among vaccinated health care workers, North Kivu, Democratic Republic of the Congo, 2021

| **Questionnaire item** | **Likert response options** | | | | | |
| --- | --- | --- | --- | --- | --- | --- |
|  | **Very much** | **Somewhat** | **Very Little** | **Not At All** | **Don’t Know/**  **Declined** |  |
|  | **n (%), N=438**  **[95% CI]** | | | | | |
| How much do you think that vaccines are good? | 258 (58.9)  [55.7–62.1] | 130 (29.7)  [27.1–32.4] | 27 (6.2)  [5.1–7.4] | 7 (1.6)  [1.1–2.4] | 16 (3.7)  [2.8–4.8] |  |
| How much do you think that vaccines are safe? | 190 (43.4)  [40.5–46.3] | 168 (38.4)  [35.8–41.0) | 36 (8.2)  [6.9–9.7] | 17 (3.9)  [3.0–5.0) | 27 (6.2)  [5.0–7.5] |  |
| How much do you think that vaccines protect against diseases? | 256 (58.4)  [55.5–61.3] | 129 (29.5)  [27.0–32.0] | 27 (6.2)  [5.1–7.5] | 14 (3.2)  [2.4–4.2] | 12 (2.7)  [2.1–3.6] |  |
| To which extent do religious leaders in your community approve of vaccination | 236 (53.9)  [50.8–56.9] | 106 (24.2)  [21.9–26.7] | 25 (5.7)  [4.3–7.5] | 14 (3.2)  [2.4–4.2] | 57 (13.0)  [10.9–15.5] |  |
| How much do other leaders in this community approve of vaccination? | 228 (52.1)  [49.1–55.0] | 118 (26.9)  [24.6–29.3] | 21 (4.8)  [3.8–6.0] | 11 (2.5)  [1.9–3.4] | 60 (13.7)  [11.4–16.4] |  |
|  | **Positively** | **Mixed** | **Negatively** | **-** | **Don’t Know/**  **Declined** |  |
| How do people in this community usually speak about vaccination? | 45 (10.3)  [8.5–12.3] | 267 (61.0)  [58.2–63.7] | 109 (24.9)  [22.5–27.5] |  | 17 (3.9)  [3.0–4.9] |  |
